# Caregiver exposure to critical events and distress in home-based palliative care in Germany a cross-sectional study using the Stressful Caregiving Adult Reactions to Experiences of Dying (SCARED) scale

**DOI:** 10.1186/s12904-019-0395-8

**Published:** 2019-01-24

**Authors:** Michael Galatsch, Holly G. Prigerson, Wilfried Schnepp, Friederike zu Sayn-Wittgenstein, Jian Li

**Affiliations:** 1 Research group “FamiLe – Family health in the life course”, Department of Nursing Science, Witten/Herdecke University, Witten, Germany; 2 Department of Nursing Science, Witten/Herdecke University, Witten, Germany; 3 Division of Geriatrics and Palliative Medicine, Department of Medicine, Weill Cornell Medicine, New York, USA; 4 Cornell Center for Research an End-Of-Life Care, Weill Cornell Medicine, New York, USA; 5 Faculty of Business Management and Social Sciences, University of Applied Sciences Osnabrueck, Osnabrueck, Germany; 6 Institute of Occupational, Social, and Environmental Medicine, Centre for Health and Society, Faculty of Medicine, University of Düsseldorf, Düsseldorf, Germany

**Keywords:** Caregiver, Palliative care, Home, Distress

## Abstract

**Background:**

Lay family caregivers of patients receiving palliative care often confront stressful situations in the care of their loved ones. This is particularly true for families in the home-based palliative care settings, where the family caregivers are responsible for a substantial amount of the patient’s care. Yet, to our knowledge, no study to date has examined the family caregivers’ exposure to critical events and distress with home-based palliative care has been reported from Germany. Therefore, we attempt to assess family caregiver exposure to the dying patient’s critical health events and relate that to the caregiver’s own psychological distress to examine associations with general health within a home-based palliative care situation in Germany.

**Methods:**

A cross-sectional study was conducted among 106 family caregivers with home-based palliative care in the Federal State of North Rhine Westphalia, Germany. We administered the Stressful Caregiving Adult Reactions to Experiences of Dying (SCARED) Scale. Descriptive statistics and linear regression models relating general health (SF-36) were used to analyze the data.

**Results:**

The frequency of the caregiver’s exposure, or witness of, critical health events of the patient ranged from 95.2% “pain/discomfort” to 20.8% “family caregiver thought patient was dead”. The highest distress scores assessing fear and helpfulness were associated with “family caregiver felt patient had enough’” and “family caregiver thought patient was dead”. Linear regression analyses revealed significant inverse associations between SCARED critical health event exposure frequency (beta = .408, *p* = .025) and total score (beta = .377, *p* = .007) with general health in family caregivers.

**Conclusions:**

Family caregivers with home-based palliative care in Germany frequently experience exposure to a large number of critical health events in caring for their family members who are terminally ill. These exposures are associated with the family caregiver’s degree of fear and helplessness and are associated with their worse general health. Thus the SCARED Scale, which is brief and easy to administer, appears able to identify these potentially upsetting critical health events among family caregivers of palliative care patients receiving care at home. Because it identified commonly encountered critical events in these patients and related them to adverse general health of family caregivers, the SCARED may add to clinically useful screens to identify family caregivers who may be struggling.

## Background

Caregivers of patients in the home-based palliative care setting especially confront stressors and experience emotional and physical burden [1–3], because the families assume responsibility for tasks and roles to provide palliative care at home [4, 5]. Furthermore, families need to deal with critical situations such as pain, insomnia, dyspnea, vomiting or other kinds of serious events [6]. Even with support from palliative care teams, the family caregivers might not be well prepared for the challenges presented as the patient approaches death [7]. Cumulative evidence has suggested that critical events are associated with many adverse physical and psychological consequences among family caregivers [8–13].

Research suggests that home-based palliative care support from professional services to family members is often not adequate and not comprehensive [14]. Given unique social and disease-related circumstances, family members have their special individual needs and perceived burdens [15]. This is particularly true for home-based palliative care in Germany, where a wide range of palliative home care services have been developed in recent decades. In 2017, there were approximately 1500 palliative home care and hospice services, and 295 specialist palliative home care services in Germany [16], covering about half of the 850,000 dying people per year, along with the family members involved [17].

One approach to assess psychosocial situations of the family at home may focus on the critical health events of terminally ill people to which family members are exposed. To date, there is an absence of assessment tools that have been adapted and validated in the German home-based palliative care context [18]. Following a comparison of available and practicable instruments, Galatsch and colleagues [18] discovered that the Stressful Caregiving Adult Reaction to Experiences of Dying (SCARED) Scale has received attention in the research of palliative care in the past years [8]. Nevertheless, the frequency and associations between these exposures and the family caregiver’s distress in terminal care among caregivers at home have not been investigated in Germany. To the best of our knowledge, our current study is the first one with emphasis on home-based palliative care services in Germany. The aim of this study was to apply the SCARED scale in Germany for assessing family caregiver exposure to critical health events in patients receiving palliative care in the home and relate these exposures to the family caregiver’s degree of distress and general health.

## Method

### Design

A cross-sectional study.

### Sample

A convenience sample was recruited in a two-step method. The palliative teams and palliative networks in the Federal State of North Rhine-Westphalia (NRW) from the directory of the Alpha (Information Centre of palliative care services for NRW) were contacted by either information materials distributed as flyers, or information sessions about the purpose of this research project. Overall 49 out of the contacted 123 palliative teams agreed to participate in our study (response rate 39.8%). The team members (physicians, nurses, etc.) of the participating 49 palliative teams disseminated the anonymous questionnaires to the family members who had a living patient whom they deemed to be at the terminal stage (i.e., within months of death) during November 2014 to November 2015, and the main person responsible family caregiver in each family answered the questionnaire. The inclusion criteria were (1) adult person aged 21 years or older; (2) family members who were responsible for caring for the patient receiving palliative care; and (3) fluent German language skills.

### Instruments

#### The stressful caregiving adult reactions to experiences of dying scale

The SCARED Scale assessed the frequency and associated fear and helplessness of ten potential distressing caregiving experiences in the provision of home care to a palliative care patient, including eight physical experiences of “severe pain or discomfort”, “inability to eat or swallow, or choking”, “vomiting”, “dehydration”, “sleeplessness”, “falling, collapsing, or passing out”, “confusion or delirium”, “other distressing experiences”, together with two psychosocial experiences of “feeling patient has had enough” and “thinking patient was dead” [8]. The family caregivers were asked to recall how often in the last month these ten experiences had happened (0: never; 1: once or twice; 2: every week; 3: every day). In a second step, the family caregivers could report the degree to which the experience evoked a sense of fear and helplessness (0: not frightened/helpless; 1: somewhat frightened/helpless; or 2: very frightened/helpless). These responses were used to calculate the SCARED event frequency score and a SCARED total score. For the SCARED event frequency score, the frequency response was summed over the ten exposures (possible range: 0–30). For the SCARED total score, the SCARED event frequency score weighted by how frightening each experience was and how helpless the experience made the caregivers felt (possible range: 0–120). Cronbach’s alpha of the original study in the USA was 0.59 for the event frequency scale and 0.77 for the total scale [8]. For this study we developed the German version of the SCARED Scale in a culturally adapted standard forward- and backward-translation process with monolingual and bilingual testing [19]. The Cronbach’s alpha was 0.59 for the event frequency scale and 0.73 for the total scale in our study. It should be noted that measures of internal consistence for assessment of life events are an imperfect assessment given it indicates the degree of co-occurrence of witnessing exposures that may be correlated, but may genuinely not need to co-occur to assess frightening, stressful exposures. More meaningful measures include correlates and outcomes of SCARED scores.

#### General health

General health was measured with a well-established 5-item subscale from the SF-36 [20]. The SF-36 was constructed to survey the health status in medical outcome studies and has been designed for the use in clinical practice and research, health policy evaluations, and general population surveys. The items were answered on a 5-point scale. For constructing the score the original 5-point scale was set from 0 to 100 following standardized instructions, higher value indicated better health [20]. Cronbach’s alpha for the general health subscale was 0.72 in our study.

### Characteristics of the participants

#### Socio-demographic information

Several socio-demographic questions were used to collect information on age, sex, marital status, education, occupation, the relationship to the patient, the number of responsible carers, and availability of professional palliative care services.

### Analyses

Descriptive statistics on socio-demographic characteristics was performed first. Responses to each item for the event frequency scale and for the total SCARED score together with mean and standard deviations (SD) were calculated. Multivariable linear regression was applied to examine the relationships between SCARED scores (total score and frequency score, respectively) and general health, controlling for the caregiver’s age, sex, relation to patient, marital status, education, occupation, number of responsible carers, and availability of professional service. Control variables were chosen on the basis of previous literature demonstrating associations between the variable and palliative caregiving / general health [8, 21, 22]. Results were shown as standardized beta coefficients, *p* < 0.05 were considered statistically significant.

## Results

### Socio-demographic characteristics

The socio-demographic characteristics of the family caregivers are shown in Table 1. In total 830 family members were contacted by the palliative team members, and 106 family caregivers agreed to join in this study and returned the questionnaires (response rate 12.7%). More than two-thirds of the family caregivers were female (67.9%) and living in partnership with the patient (79.3%). The mean age of the family caregiver was 58.3 (range 33–82) years. The family members who cared for the patients were predominately close family members like spouses or children (83.0%). In 77.3% of all cases, two or more persons shared the responsibility for the patients. Most of the family caregivers (75.5%) had no general qualification of university entrance (> 12 years of schooling) according to the German education system. Nearly half of the family caregivers had a job while caring for the patients. Professional palliative services, such as ambulatory palliative service and outpatient hospice care, were available to most of the families (84.9%). The mean score of general health in our sample was 42.1, which was lower than the population-based average level (65.4–67.6) using the same measure, among German people with the same age and sex distribution [23].

**Table 1:** Socio-demographic profile of family caregivers

Characteristics		n	%	Mean	SD
age				58.3	12.5
	missing	9	8.5		
sex	female	72	67.9		
	male	34	32.1		
relation to Patient	Spouse/partner/ Child	88	83.0		
	Other	16	15.1		
	missing	2	1.9		
marital status	single	21	19.8		
	partnership	84	79.3		
	missing	1	0.9		
education	<= 12 years of schooling	80	75.5		
	> 12 years of schooling	26	24.5		
responsible carers	only one person	22	20.8		
	two persons	72	67.9		
	more than two persons	10	9.4		
	missing	2	1.9		
occupation	yes	49	46.2		
	no	45	42.5		
	missing	12	11.3		
professional service	palliative care service	90	84.9		
	health service without palliative focus	14	3.2		
	missing	2	1.9		
general health				42.1	12.7

### The SCARED prevalence and frequency score

The highest prevalence of critical physical caregiving events were *Severe pain/ discomfort* 95% (*n* = 101) (Table 2), followed by *insomnia* 52.8% (*n* = 56) and *confusion, delirium* 51.8% (*n* = 55). Notably, the frequency of one psychosocial event, *family caregiver felt patient “had enough”*, was as high as 53.7% (*n* = 57). The means for SCARED total score and frequency score were 19.4 and 7.7, respectively.

**Table 2:** SCARED overall event prevalence among caregivers and summary scores of SCARED Scale and general health

Overall prevalence of critical events	n (%)
severe pain/discomfort	101 (95,2)
unable to eat or swallow/choking	45 (42.5)
vomiting	48 (45.3)
dehydration	29 (27.3)
insomnia	56 (52.8)
falling, collapsing, passing-out	46 (43.4)
confusion, delirium	55 (51.8)
other events	21 (19.8)
family caregiver felt patient “had enough”	57 (53.7)
family caregiver thought patient was dead	22 (20.8)
Summary scores	Mean (SD)
SCARED: total score (range 0–120)	19.4 (13.3)
SCARED: event frequency score (range 0–30)	7.7 (4.1)

Table 3 shows that the frequently mentioned daily adverse events were *Severe pain/ discomfort* (37.7%, *n* = 40) and *Unable to eat or swallow/choking* (12.3%, *n* = 13). *Severe pain/ discomfort* (34.9%, *n* = 37), *confusion, delirium* (22.6 *n* = 24) and *family caregiver felt patient “had enough”* (16% *n* = 17) were the most weekly frequent events. Regarding less frequently happened events (once/twice), *Insomnia* (37.7% n = 40), *Family caregiver felt patient “had enough”* (34.9% n = 37) and *Falling, collapsing, passing-out* (32.1 *n* = 34) were reported.

**Table 3:** SCARED Frequency and Distress Scores

SCARED Frequency and Distress scores (*n* = 103)	Never (frequency score = 0)	Once/Twice (frequency score = 1) (%)		Weekly (frequency score = 2) (%)		Daily (frequency score = 3) (%)		Mean Fear score (SD)		Mean Helplessness score (SD)	
severe pain/discomfort	3	24	(22.6)	37	(34.9)	40	(37.7)	1.15	(.70)	1.38	(.66)
unable to eat or swallow/choking	59	20	(18.9)	12	(11.3)	13	(12.3)	1.33	(.56)	1.20	(.76)
vomiting	56	27	(25.5)	18	(17.0)	3	(2.8)	1.19	(.70)	1.33	(.66)
dehydration	75	17	(16.0)	2	(1.9)	10	(9.4)	1.17	(.85)	.83	(.71)
insomnia	48	40	(37.7)	6	(5.7)	10	(9.4)	.66	(.67)	.71	(.76)
falling. Collapsing. passing-out	58	34	(32.1)	12	(11.3)	–	–	1.22	(.51)	1.30	(.66)
confusion. Delirium	49	23	(21.7)	24	(22.6)	8	(7.5)	1.44	(.66)	1.40	(.71)
other events	79	5	(4.7)	5	(4.7)	11	(10.4)	.33	(.48)	.67	(.86)
family caregiver felt patient “had enough”	47	37	(34.9)	17	(16.0)	3	(2.8)	1.68	(.47)	1.63	(.56)
family caregiver thought patient was dead	81	18	(17.0)	4	(3.8)	–	–	1.59	(.80)	1.45	(.86)

Mean fear score (range 0–6): frequency of event × fear score (0 = not frightened, 1 = somewhat frightened, 2 = very frightened) among those who have a reported frequency > 0

Mean helplessness score (range 0–6): frequency of event × helplessness score (0 = not helpless, 1 = somewhat helpless, 2 = very helpless) among those who have a reported frequency > 0

### The SCARED distress score

In general, the highest fear and helplessness scores were found to be due to exposure to two psychosocial events, *family caregiver felt patient “had enough”* (fear score 1.68 and helplessness score 1.63) and *Family caregiver thought patient was dead* (fear score 1.59 and helplessness score 1.45). Exposure to physical distressing events exerted relatively lower levels of fear and helplessness, for instance, fear score was 0.66 and helplessness score was 0.71 due to *insomnia*.

### Associations of SCARED scores with general health

As seen in Table 4, Model 1 represents results for SCARED total score and Model 2 represents results for SCARED frequency score. Both SCARED total score (beta = − 0.377, *p* = 0.007) and exposure frequency score (beta = − 0.408, *p* = 0.025) were negatively associated with caregivers’ perception on their own general health. The socio-demographic factors did not show any significant associations with general health.

**Table 4:** Associations with general health by multivariable linear regression

	Model 1 (for SCARED total score) general health		Model 2 (for SCARED frequency score) general health	
adjusted R Square	.112		.083	
	standardized beta coefficient	*p*-value	standardized beta coefficient	*p*-value
(Constant)				
SCARED total score	**−.377**	**.007**	N/A	N/A
SCARED frequency score	N/A	N/A	**−.408**	**.025**
Age in years	.149	.505	.139	.939
People who principally involved in care 1 = only one	.063	.617	.074	.560
Relationship status in 2 groups	−.137	.365	−.164	.285
Highest School education 1 = no general qualification for university entrance	.148	.178	.158	.157
Sex 1 = female	.121	.363	.115	.414
Occupation 1 = yes	−.071	.562	−.008	.950
Relation to Patient 1 = close family	.115	.382	.061	.642
Professional service 1 = palliative service	.049	.720	.047	.735

## Discussion

The objective of this study was to examine the frequency of stressful health events of German home-based palliative care patients and how these events related to fear and helplessness as well as the general health, or quality of life, of the family caregiver. Overall, the families in our sample were facing several critical health events in the care of the dying patient at home that were distressing to them. These exposures evoked a certain level of fear and helplessness in the family members. Compared to the original study from the USA by Prigerson [8], both SCARED total score and frequency score were relatively lower in our study (total score 23.2 vs. 19.4, frequency score 10.6 vs. 7.7). When looking at each critical event closely, we found the family caregivers in our sample reported overall more exposures to *“severe pain/discomfort”*, *“falling, collapsing, passing out”*, *“vomiting”* and “*insomnia*”, whereas other exposures were less frequently reported in our study. With respect to fear and helplessness, exposure to two psychosocial events, *family caregiver felt patient “had enough”* and *family caregiver thought patient was dead* contributed most in our study, but the USA study found *“falling, collapsing, passing out”* also produced much fear and helplessness.

The differences can be explained in a couple of ways. First, Prigerson’s study was conducted in a hospice inpatient setting and our study was conducted in a home-based palliative care setting. The patient profile of the study participants may have been different. Although we did not have direct information to compare the two studies, it was of interest to note that caregivers reported more daily-based frequency of critical events in the USA Prigerson’s study; by contrast, data from our study revealed that a higher prevalence of less frequent exposure to these caregiving stressors (weekly or once/twice) were reported more often. Despite the fact that the daily frequency of critical health care events in Prigerson’s study was higher, we found a trend toward higher mean scores of fear and helplessness on most critical events in our study. This could also be attributed to the differences between both settings. The family caregivers may have greater support for care of the patients in the hospice settings. In home-based palliative care, the demands on family members are greater and they are less trained and/or prepared for caring for a dying person [7, 24–26]. Second, differences between healthcare systems and the development of palliative care services might also be different in our findings and those of Prigerson et al. [16, 27, 28]. As mentioned earlier, home-based palliative care in Germany has developed considerably in the past years, and is covered by the universal medical insurance to everyone in the country [17]. Perhaps differences in acceptance and coverage in palliative care services between Germany and the USA account for differences between SCARED scores. Nevertheless, our results are in line with previous studies [8, 29], both total and frequency scores exerted negative effects on general health of the family caregiver.

Hudson and colleagues [30] suggested the systematically implementation of assessments as a standard in the palliative care practice. Regular use of assessment instruments can lead to improvement of the quality of care of patients and family outcomes [31–34]. Therefore, a combination of systematically applied instruments and individual-tailored offers of consultation from the palliative services could be of help to better integrate and include the families during the care process, and at the same time to meet their needs better. For this purpose, the SCARED Scale, due to its unique properties (brief and easily administered instrument), could serve as a screening tool in palliative care to identify distressed family caregivers who are in need of extra or further support. Also, this instrument could be systematically applied to obtain an initial and continuous assessment about the frequency and burden of distressing events within the home-based palliative care situation. Future research for individual changes of the distress among family caregivers during the entire palliative care situation, taking different patient characteristics into account, is needed.

To our knowledge, this is the first study to assess caregiver exposure to distress with home-based palliative care in Germany; also, this is the first study that we know of to use the SCARED Scale out of the USA. However, several limitations need to be considered. First, the response rate of our study was not high. The potential reasons might be low acceptance of palliative research and/or high workloads of the contacted palliative teams in Germany. By contrast, a recent study found that family caregivers in Australia appreciated the opportunities to participate in palliative research, indicating potentially cross-cultural difference [35]. Thus, non-response bias could not be ruled out when interpreting the current findings. However, the sample size of our study seemed to be adequate for generating meaningful findings, compared to previous studies [8]. Second, due to the nature of convenience sampling with two steps, it is impossible for us to yield a random sample of family caregivers; in addition, we had little information how the palliative teams distributed the questionnaires to the target population. Thus, the power to generalize our research findings to other settings is limited. Third, due to the nature of paper-based survey with self-reported data, common method bias and recall bias on the observed associations cannot be ruled out, given the fact that family members reported patients’ symptoms significantly worse than professionals [36]. Finally, some potentially relevant factors which might relate to caregivers’ general health were not included in our current study, for example, perceived burden of care or profile (type and severity) of patients’ condition.

## Conclusion

In conclusion, the findings of our study suggest that the family caregivers with home-based palliative care in Germany are facing a number of critical health events of the dying patients, resulting in fear and helplessness; both exposure and distress are associated with poor health. In addition to the initial study in hospice settings from the USA, we found that the SCARED Scale could be applied as a screening tool in home-based palliative care setting to identify distressed family caregivers with a potential need of extra or further support. Considering the negative effects of caring for a dying relative on individuals, families, and societies, future research, with a well-tested instrument in the palliative practice assessing caregivers’ psychosocial situation, such as the SCARED scale, is recommended or suggested within and beyond American and German contexts.

## Abbreviations

NRW: North Rhine-Westphalia
SCARED: Stressful Caregiving Adult Reaction to Experiences of Dying
SF-36: 36-Item Short Form Health Survey
USA: United States of America
